# Scalable Gastric Resident Systems for Veterinary Application

**DOI:** 10.1038/s41598-018-30212-3

**Published:** 2018-08-07

**Authors:** Alison Hayward, Taylor Bensel, Hormoz Mazdiyasni, Jaimie Rogner, Ameya R. Kirtane, Young-Ah Lucy Lee, Tiffany Hua, Ambika Bajpayee, Joy Collins, Shane McDonnell, Cody Cleveland, Aaron Lopes, Aniket Wahane, Robert Langer, Giovanni Traverso

**Affiliations:** 1 The David H. Koch Institute for Integrative Cancer Research, Massachusetts Institute of Technology, Cambridge, Massachusetts 02139 USA; 2 Department of Chemical Engineering, Massachusetts Institute of Technology, Cambridge, Massachusetts 02139 USA; 3 Division of Gastroenterology, Brigham and Women’s Hospital, Harvard Medical School, Boston, Massachusetts 02115 USA; 4 Institute for Medical Engineering and Science, Massachusetts Institute of Technology, Cambridge, Massachusetts 02139 USA; 5 Division of Comparative Medicine, Massachusetts Institute of Technology, Cambridge, Massachusetts 02139 USA; 6 Department of Bioengineering, Northeastern University, Boston, Massachusetts 02115 USA

## Abstract

Gastric resident dosage forms have been used successfully in farm animals for the delivery of a variety of drugs helping address the challenge of extended dosing. Despite these advances, there remains a significant challenge across the range of species with large variation in body size. To address this, we investigate a scalable gastric resident platform capable of prolonged retention. We investigate prototypes in dimensions consistent with administration and retention in the stomachs of two species (rabbit and pig). We investigate sustained gastric retention of our scalable dosage form platform, and in pigs show the capacity to modulate drug release kinetics of a model drug in veterinary practice, meloxicam, with our dosage form. The ability to achieve gastric residence and thereby enable sustained drug levels across different species may have a significant impact in the welfare of animals in both research, agricultural, zoological, and clinical practice settings.

## Introduction

Prolonged drug release systems have the capacity to minimize human intervention in both livestock and pets, and thereby maximize therapeutic efficacy while minimizing potential discomfort and stress to the animal subject. Some of the first descriptions of veterinary sustained release dosage forms are found in patent applications from the 1960s and 1970s^1–3^. These describe pellets or boluses with particular weight or densities that are designed to reside in the rumeno-reticular space of cattle or sheep for extended periods while releasing therapeutics such as antibiotics or nutrients. Various other elegant strategies have been employed for gastric retention in cattle. These include systems that reconfigure their shapes to avoid regurgitation, osmotic pumps and gelling agents^4–6^. These systems have been used for a variety of therapeutic applications (such as preventing bloat, infectious diseases and other disorders), as well as diagnostics to monitor the gastric environment^7–10^.

In smaller species of animals like dogs, dosage forms have been shown to remain in the gastric space for at least 24 hours for controlled release of drugs with a narrow absorption window to improve therapeutic outcomes, e.g., furosemide and levodopa^11^. El-said^12^ described a gellan gum superporous hydrogel hybrid system, evaluated the release of baclofen, and investigated retention in the gastric space for at least 6 hours with improved extended release of drug and prolonged plasma profiles when compared to twice daily administration. The challenge with dosage forms in canine stomachs are related to the retention time as they are known to have rapid gastric emptying and rapid intestinal transit times and relatively strong migrating motor complexes when compared to humans, pigs or rabbits^13,14^. Cargill^15^ showed that device shape, size and flexibility affect the gastric resident times and investigated a retention time of 24 hours in beagle dogs.

In other monogastric species like pigs, there has been considerable work in our group that has investigated resident times of up to 2 weeks^16–18^. Pigs make a good model due to their availability and physiological and anatomical similarities to humans^19–21^, however, they have a much slower gastrointestinal transit time than dogs or humans^13^. In smaller monogastric herbivorous animals, such as rabbits, where gastric emptying is slower than dogs and can be greater than 24 hours^13^, gastric resident systems may be more amendable to prolonged resident times for drug release. Khan and Dehghan^22^ looked at increased bioavailability of atorvastatin calcium when floating tablets were administered and drug released over a duration of 6 hours in rabbits. Thakar^23^ reported floating retention times of 12 hours in rabbits and improved bioavailability of baclofen compared to the marketed sample.

In other species, like non-human primates, systems that monitor gastric pH and residence times have been described^24^ but to our knowledge no resident systems have been described for drug release over time.

**Figure 1:**
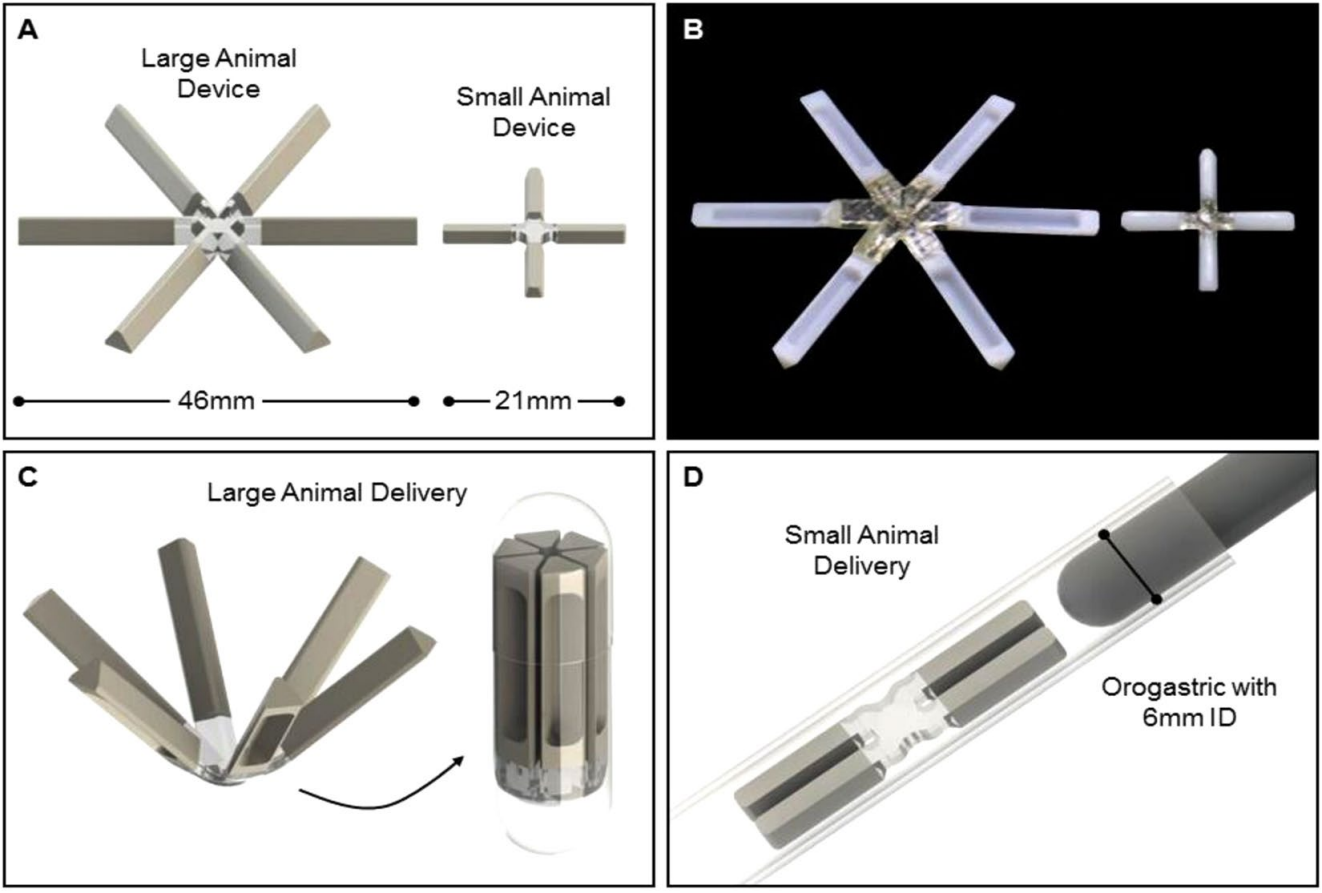
Design and administration of oral gastric retentive device for meloxicam. (**A**) Schematic of gastric resident dosage forms scaled for rabbits and pigs. (**B**) Representative solid arm dosage forms pre-administration. (**C**) Illustration of large animal dosage form folding and being encapsulated, enabling oral administration. (**D**) Diagram showing small animal dosage form folding and being delivered via a 6 mm orogastric tube.

There is no ideal animal model which perfectly recapitulates human gastrointestinal characteristics including motility^19^. Each animal possesses unique challenges based on its size, anatomy and physiology that inform different retention characteristics and drug profiles. To explore these unique challenges and develop potential long term controlled drug release systems for small and larger animal species, we chose two common species to investigate the potential for a gastric resident dosage form. Prototypes compatible with the two anatomies were developed for orogastric delivery (Fig. 1). Controlled drug release using these forms was investigated with meloxicam.

Meloxicam, a selective cyclooxygenase-2 inhibitor, is a non-steroidal anti-inflammatory drug used as an analgesic, antipyretic and anti-inflammatory in acute and chronic settings depending on the species^25–35^. The drug is formulated for oral and injectable routes of administration for repeat administration or sustained release. We aimed to investigate the incorporation of this drug as a model agent into our gastric resident systems for oral administration to investigate sustained release over three days in the pig.

We report a gastric resident dosage form that can be maintained in the stomach for at least 3 days without compromising drug stability and can safely pass without causing injury to the animals. We investigate that in both rabbits and pigs, dosage forms reside in the stomach for at least 3 days, release drug at a consistent rate in the pig and in both species pass without issue. This rabbit model is the first known small animal model to investigate retention of dosage forms such as these in the stomach for at least 3 days, with safe transit out of the gastrointestinal tract. Application of these dosage forms in large and small animals could be useful for short or long term administration of drugs orally for clinical or research applications. This study also investigates the usefulness of the pig and rabbit as models for extended delivery of drugs from gastric resident systems. Although we investigated meloxicam, other drugs could be incorporated for extended release.

## Materials and Methods

### Materials

Meloxicam was obtained from Hangzhou Hysen Pharma Co. Ltd. (Hangzhou, China). Poly(dimethyl siloxane) (PDMS) negative molds for manufacturing dosage forms were purchased from Proto Labs (MN, USA). Povidone K25 and Kollidon CL were obtained from BASF (NJ, USA). All other chemicals were obtained from Sigma Aldrich (MO, USA).

### High performance liquid chromatography (HPLC)

Drug concentration was analyzed using Agilent 1260 Infinity HPLC system equipped with Model 1260 quaternary pump, Model 1260 Hip ALS autosampler, Model 1290 thermostat, Model 1260 TCC control module, and Model 1260 diode array detector. The output signal was monitored and processed using Agilent ChemStation software. Chromatographic separation was carried out on a 50-mm × 4.6-mm EC-C18 Agilent Poroshell 120 analytical column with 2.7-µm spherical particles, maintained at 40 °C.

For samples in MeOH: DMSO solution, the optimized mobile phase consisted of a gradient elution of methanol: water (pH 3.5 adjusted with 0.1% formic acid) (0 minute (min), 30:70 v/v; 4 min, 30:70; 4.5 min, 90:10; 7 min, 90:10; 7.5 min, 30:70) at flow rate of 1.1 mL/min over a 12-min run time. The injection volume was 20 µL, and the UV detection wavelength of 360 nm was selected.

For samples in simulated gastric fluid (SGF, which consists of 0.2% w/v sodium chloride, 0.7% w/v hydrochloric acid, deionized water, pH 1.2), the optimized mobile phase consisted of a gradient elution of methanol: water (pH 3.5 adjusted with 0.1% formic acid) (0 min, 30:70 v/v; 3.5 min, 70:30; 6.5 min, 70:30; 7.5 min, 30:70) at flow rate of 1.0 mL/min over a 10-min run time. The injection volume was 50 µL, and the UV detection wavelength of 360 nm was selected.

### Liquid chromatography–tandem mass spectroscopy (LC-MS)

Ultra-performance LC (UPLC) separation was carried out on a Waters UPLC aligned with a Waters Xevo TQ-SMS mass spectrometer (Waters Corp., USA). MassLynx 4.1 software was used for data acquisition and analysis. LC separation was performed on an Acquity UPLC Charged Surface Hybrid C18 (50 mm × 2.1 mm; particle size, 1.7 mm) at 50 °C. The mobile phase consisted of acetonitrile, 0.1% formic acid, 10 mM ammonium formate solution and flowed at a rate of 0.6 ml/min using a time and solvent gradient composition. The initial composition (80% aqueous) was held for 0.5 min, then over the next 2.0 min the gradient was brought to 0% aqueous and held for additional 1.0 min. At 0.01 min shifted back to 80% and held constant until the end of the run for column equilibration. The total run time was 4.0 min, and sample injection volume was 2.5 µL. The mass spectrometer was operated in the multiple reaction-monitoring mode. Sample introduction and ionization was conducted with an electron spray ionization (ESI) in the positive-ion mode.

Stock solutions of meloxicam and an internal standard, piroxicam, were prepared in methanol at a concentration of 500 µg/mL. A 9-point calibration curve ranging from 5 to 2500 ng/mL was prepared. Quality control samples were prepared by a similar procedure using an independent stock solution at three concentrations (5, 25, and 250 ng/mL). To prepare the samples, 200 µL of internal standard in methanol was added to 100 µL of sample solution to cause precipitation. Samples were vortexed and sonicated for 10 min and then centrifuged at 15000 RPM for 10 min. An aliquot of 200 microliters of solution was pipetted into a 96-well plate containing 200 µL of water. Finally, 2.5 µL of sample was injected into the UPLC-ESI-MS system for analysis.

### Determining the stability of meloxicam in SGF

Meloxicam was added to SGF at a concentration exceeding its solubility in the solvent. The suspension was sonicated in a water bath and filtered using a 0.45 µm PTFE filter. The filtrate was incubated at 37 °C, and at various time points aliquots were collected and stored at −20 °C until further analysis. Drug concentration was assessed using HPLC.

### Evaluating the heat stability of meloxicam

To assess the heat stability of meloxicam, the drug was weighed into scintillation vials and incubated in a convection oven. Stability tests were performed at 70 °C and 120 °C. At different time points, the vials were removed from the oven, allowed to cool and the drug was dissolved in 90:10 MeOH/DMSO mixture to make 100 µg/mL solution. The concentration of the samples was determined via HPLC. The time-points were taken with three replicates each.

### Manufacturing of gastric resident dosage forms

The design of the dosage form used in these studies is as described by Bellinger^16^. It consists of an elastomeric core interfaced with rigid arms to allow its folding, either into a capsule shell or for passage down an orogastric tube, and expansion in the stomach upon dissolution of the capsule shell or exit from the tube. Two different sizes of dosage forms were made, one size for large animals and one size for small animals. (Fig. 1A,B).

### Manufacturing of dosage forms for large animal studies

**Figure 2:**
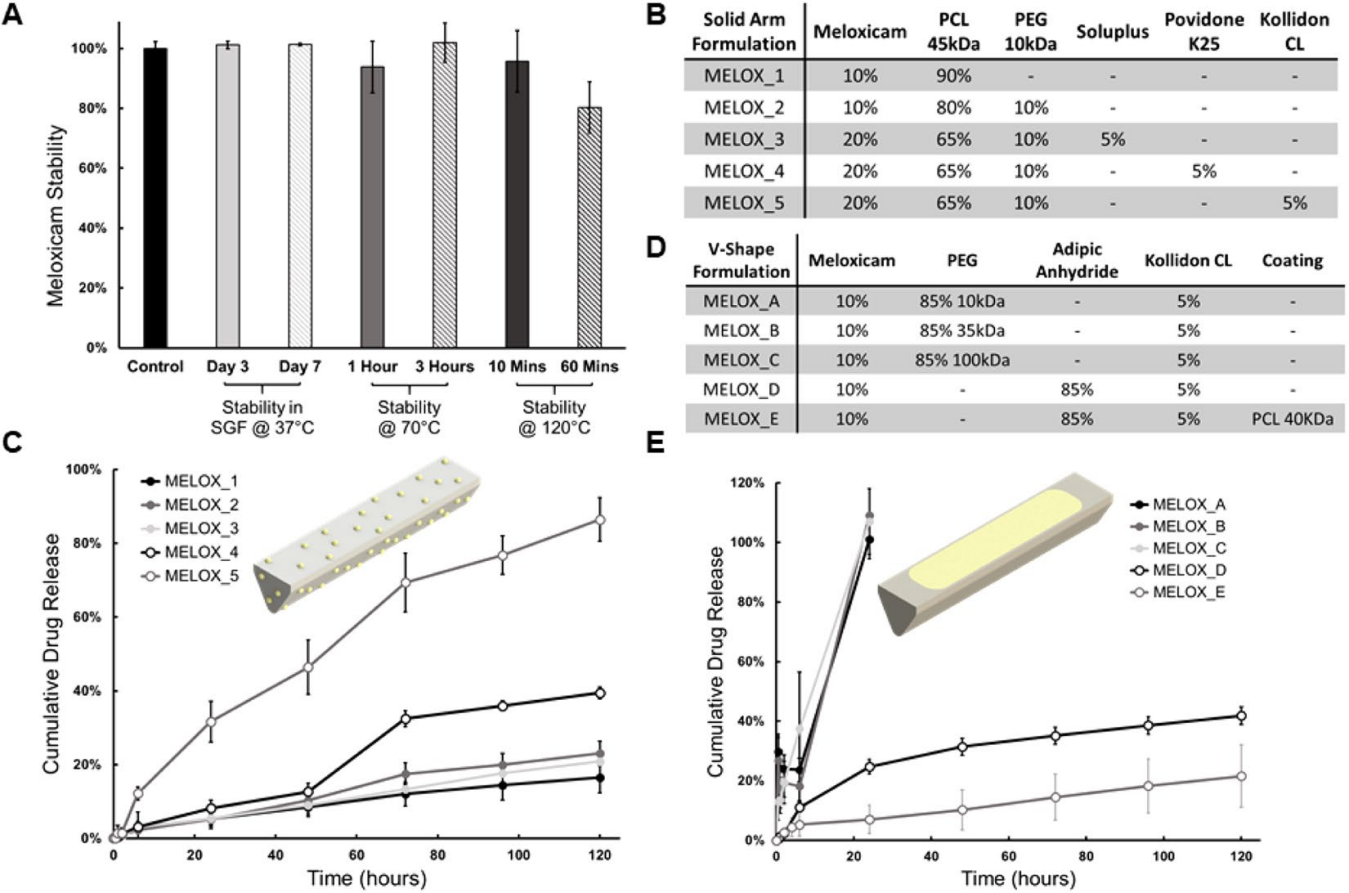
*In vitro* stability and release of meloxicam. (**A**) Heat stability of meloxicam and stability in SGF over time. (**B**) Compositions of solid arm formulations by weight percent used for *in vitro* and *in vivo* analysis. (**C**) *In vitro* release of meloxicam from solid arms in 3% Kolliphor RH 40 over 5 days. For all *in vitro* release data, each data set reflects an average of the percent of initial drug released at each time point for triplicate sample preparations and error bars denote the standard deviation of the three percentage measurements. (**D**) Compositions of v-shape arm formulations by weight percent used for *in vitro* and *in vivo* analysis. (**E**) *In vitro* release of meloxicam from v-shape arms in 3% Kolliphor RH 40 over 5 days.

The dosage forms used in the pigs differed in size, composition and arm design. The first design in the pigs consisted of a monolithic material, made of a polymer blend with drug dispersed throughout the polymer matrix. In this paper, we refer to these dosage forms as *solid arm dosage forms* (Fig. 1A) and they were made of the polycaprolactone (PCL) composition described below (these are abbreviated MELOX_1–5 depending on composition, see Fig. 2B). The arms of the second design of the dosage form contained two parts; an outer casing manufactured with a drug-polymer matrix placed in V-shaped cavity. This version of the dosage form is referred to as the *v-shaped arm dosage form* (these are abbreviated MELOX_A – E, see Fig. 2D). The MELOX_VI formulation was made with four arms filled with MELOX_C and two arms filled with MELOX_D which resulted in 66.6% of the meloxicam coming from MELOX-C and 33.3% from MELOX-D, see Fig. 4D). Both designs tested in pigs were 46 mm in diameter.

The solid arm dosage forms utilize a PCL based polyurethane thermoset elastomeric core described by Bellinger^16^ and arms made of a linear 80 kDa PCL (referred to as PCL/PCL). To prepare the dosage form, pellets of 80 kDa PCL were placed into a custom PDMS mold and melted in an oven at 90 °C. After cooling, the central core was cut out, leaving just 80 kDa PCL arms in the mold. The thermoset elastomer was then cured in the void space of the mold at 70 °C for 48 hours and the arms were trimmed to leave approximately 3 mm of each remaining.

To prepare the arms of the dosage forms, drug-polymer mixture was extruded and injection molded using a 5 mL Xplore twin screw micro-compounder and 5.5 mL laboratory injection molder (Xplore Instruments, Sittard, Netherlands). Compounding and extruding were executed at 65 °C with a screw speed of 75 RPM. The injection molder barrel and plunger were heated to 65 °C and the mold temperature was 22 °C. Material was injected using a ramp function that controlled the pressure into a standard die to generate the bending bars and a custom-made stainless steel die to create the arms. The pressure started at 1.5 bar for one second, was ramped up to 3 bar over one second, and was held at 3 bar for an additional 5 seconds.

The injection-molded arms were then placed in the same mold with the trimmed elastomeric core in the middle and heated at 70 °C in an oven for 1 hour to facilitate bonding. While still melted, three sequential markers (2 mm steel ball bearings) were placed in each arm to enable radiographic visualization of the dosage forms *in vivo*. The mold was then removed from the oven and cooled to room temperature. The dosage forms were finally removed from the molds and stored at room temperature until further use.

The *v-shaped arm dosage form* were comprised of Sorona 3015 G NC010 (DuPont, DE, USA) arms, a fiber reinforced thermoplastic, and thermoplastic polyurethane central elastomers made from Elastollan 1185A10 (BASF, NJ, USA). The arms were manufactured by feeding Sorona 3015 G NC010 pellets into the micro-compounder and injection molding the molten material. Compounding and extruding were executed at 265 °C with a screw speed of 50 RPM. The injection molder barrel and plunger were heated to 265 °C and the mold was heated to 100 °C. Material was injected using a ramp function into a custom arm shaped die. The pressure started at 3 bar for one second, ramped up to 4.5 bar over one second, and was held at 4.5 bar for an additional 5 seconds. Post injection molding, a v-shaped pocket was milled into the arms which were then interfaced with a central core of Elastollan 1185A10 as laid out by Kirtane^17^. Drug-polymer mixtures were filled into the v-shaped pockets at 140 °C as described previously. Briefly, a mixture containing 60% poly(ethylene glycol) (35 kDa) or poly(adipic anhydride), 10% Kollidon CL, and 30% meloxicam was heated to 140 °C in a glass vial. After the principal component of the mixture melted, the contents were thoroughly mixed and transferred to the v-shaped arms using a spatula. After the mixture melted again in the arms, the dosage forms were removed from the oven to cool to room temperature.

We also prepared V-shaped arms coated with a polymer to modify drug release. A coating solution poly(caprolactone) (40 kDa) in acetone was prepared by dissolving the polymer in solvent at ~50 °C and at a concentration of 12%w/w. Coating was applied by dipping the arms in the polymer solution 5 times for 5 seconds each.

### Manufacturing of dosage forms for small animal studies

All rabbit dosage forms spanned 21 mm in diameter. The two compositions of the small animal dosage forms differed only in the central elastomeric material.

PCL/PCL dosage forms were composed of PCL arms interfaced with the same PCL-based polyurethane thermoset used in large animals. These were manufactured using the same process to cure the elastomer to the arms, scaled down in size for smaller species.

PCL/PU dosage forms, spanning 21 mm, was also manufactured using the same process as stated above in “*Manufacturing of dosage forms for large animal studies”* using 1 mm steel ball bearings instead of the 3 mm.

### Three Point Bending Mechanical Testing

The structural stability investigation of the formulations was performed on an Instron 5943 (Instron, MA, USA) according to ASTM D5023 standards. Drug-loaded polymer matrices were injection molded as described before. Injection molded bars (63 mm × 13 mm × 3.25 mm) were analyzed using the Instron three-point bending apparatus. A digital micrometer was used to measure the width and thickness of specimens prior to testing. Three samples of each formulation were tested at a rate of 15 mm/min until failure. The measured load and deflection were converted and graphed to generate a flexural stress versus flexural strain plot for each sample. From these, the average ultimate flexural stress was calculated for each formulation along with the average flexural strain at which the ultimate stress was measured.

### Evaluation of the *In vitro* release of meloxicam

We tested the release of meloxicam from a variety of polymer-based matrices molded in the shape of arms for the *solid arm dosage form* or *v-shaped arm dosage form*. In both cases, drug-loaded polymer matrices were cast as arms as described in the previous section. For the *in vitro* drug release studies, only the drug-loaded arms were used, not the entire gastric resident dosage form.

To perform the drug release study, drug-loaded arms were placed in 20 mL SGF containing 3% Kollidon RH 40 in an incubator shaker at 150 RPM and 37 °C (New Brunswick Innova 44, Eppendorf, USA). At various times, a part of the release medium was collected and stored at −20 °C until further analysis. At the end of the study, the samples were thawed and analyzed using HPLC.

### *In vivo* evaluation of gastric retention of the dosage form

All *in vivo* experiments were approved by the Committee on Animal Care, at MIT, in accordance with all local, state and federal regulations. The different dosage form designs were created as previously described for the large and small animal models as depicted in Fig. 1A,B. Pigs were chosen as the large animal model due to the similar gastric anatomy to humans^21,36^ making it an ideal model for translational studies. Rabbits were chosen as a small animal model as a good representative of a non-rodent but small animal model.

**Figure 3:**
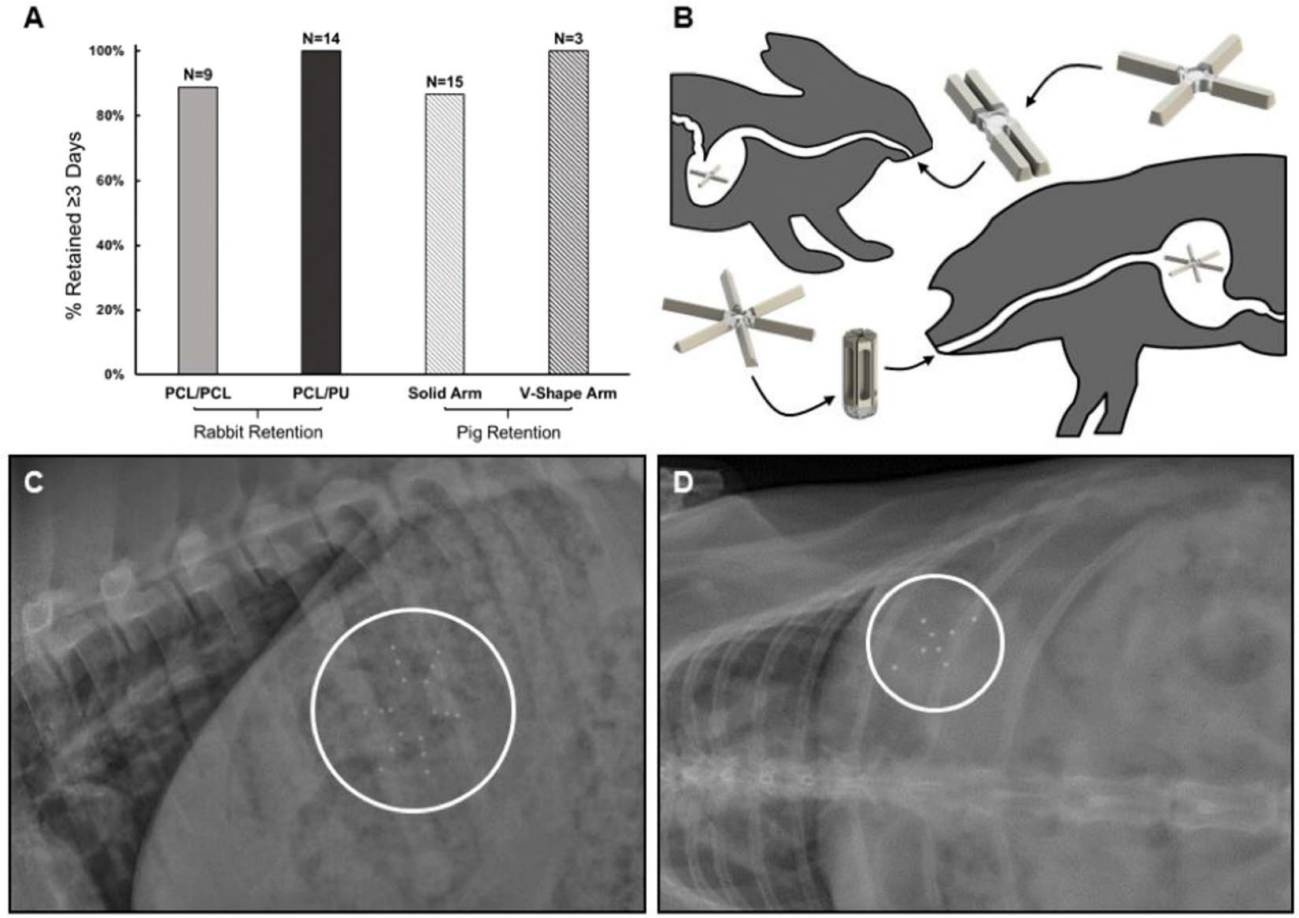
Gastric retention of dosage forms. (**A**) Percentage of intact dosage form retained in the stomach of rabbit and pig models ≥3 days. (**B**) Illustration of dosage form delivery and dosage form retention in rabbit and pig models. (**C**) Representative lateral radiograph showing the dosage form residing in a pig. (**D**) Representative dorsoventral abdominal radiograph showing the dosage form residing in a rabbit.

Female, Yorkshire pigs, 35–50 kg, were obtained from a local source and were housed in conventional pens. They were fed twice daily and had ad libitum access to water. Pigs were anesthetized with Telazol (tiletamine/zolazepam, 5 mg/kg), xylazine (2 mg/kg), and atropine (0.05 mg/kg) administered via intramuscular injection. An endoscope with an overtube was placed into the stomach via the esophagus. A 000 gelatin capsule containing the folded large animal dosage was placed down the overtube, using the endoscope to confirm delivery into the gastric cavity form (Figs 1C and 3B). Each dosage form was given to three animals which based on experience has yielded informative results for initial pharmacokinetic analysis^17^ (see Figure S1).

Female, New Zealand White rabbits, 3–4 kg, were anesthetized with ketamine (35 mg/kg) and xylazine (5 mg/kg) administered via intramuscular injection. An 18 French soft rubber orogastric tube was place via the esophagus into the stomach. The small animal dosage form was folded and placed into the orogastric tube (Figs 1D and 3B). A flexible stylet was used to pass the dosage form into the stomach. For numbers of animals in each group, refer to table in Table S1.

In both species, radiographs were taken to confirm correct delivery to the gastric space and repeated every other day until the dosage form had passed. Animals were evaluated twice daily for clinical signs of obstruction. No complications were associated with the delivery or passage of the dosage form.

### *In vivo* evaluation of pharmacokinetics of meloxicam

To characterize plasma pharmacokinetics of meloxicam in pigs, blood was taken from the superficial epigastric veins on the ventral abdomen at 0 min (prior to dosing), 15 min, 30 min, 1 hour, 2 hours, 6 hours and then daily for 7 days. Blood samples were collected in BioLegend Vacutainer gold top tubes (Becton, Dickinson and Co., NJ, USA). The blood samples were centrifuged at 3000 RPM and 4 °C for 10 min to separate the plasma, which was stored at −80 °C until further analysis. Drug concentration in the plasma was analyzed using liquid chromatography-tandem mass spectroscopy (LC-MS).

## Results

### Stability of meloxicam at elevated temperatures and acidic pH

The dosage form described in this study is retained in the stomach and releases drug in the gastric environment over a prolonged duration. Hence, it was imperative that the drug used in our studies was stable at an acidic pH. HPLC-based analysis revealed that meloxicam was stable in SGF for up to 7 days.

As the dosage form was manufactured at a high temperature, we tested the heat stability of the drug. We observed minimal degradation upon heating to 70 °C for 3 hours. There was no drug degradation even upon heating to 120 °C for 10 min; however, loss in drug stability was observed if maintained at this temperature for a prolonged period (Fig. 2A).

### Drug release and mechanical stability of PCL-based solid arms

We synthesized various PCL-based formulations of meloxicam differing in the amount of drug loaded in these systems and containing various hydrophilic polymers such as poly(ethylene glycol), Soluplus, Povidone K25 and Kollidon CL. The compositions of these formulations are described in Fig. 2B.

Drug release from the formulation that contained only PCL and meloxicam (MELOX_1) progressed at a zero-order rate (Fig. 2C) and about 10–15% of the drug was released over 5 days. Addition of poly(ethylene glycol) (MELOX_2) or Soluplus (MELOX_3) did not have any significant influence on drug release. Interestingly, inclusion of Povidone K25 (MELOX_4) and Kollidon CL (MELOX_5) increased the overall amount of drug released by about 3- and 8-fold respectively. Formulations MELOX_2, MELOX_3, and MELOX_5 were selected based on these results to see if they would show similar trends *in vivo*.

We then explored the effect of excipients on the mechanical properties of the arms given the recognized importance of mechanical integrity on effective gastric retention^16^. Any formulation that showed large reduction in flexural strength of the arms could result in poor gastric retention. Arms made of PCL alone displayed a maximum flexural stress of 37.1 ± 3.0 MPa (Table 1). This served as a baseline for comparison with the polymer drug formulations. The addition of meloxicam and a small percentage of poly(ethylene glycol) to PCL (MELOX_1) resulted in a slight increase in the maximum flexural stress (40.1 ± 2.1 MPa). The maximum flexural stress of the arms containing a higher concentration of poly(ethylene glycol) with Soluplus as well as the arms containing Kollidon CL (MELOX_5) resulted in lower maximum flexural stresses, 30.50 ± 0.59 and 27.47 ± 2.08 respectively. Although these were roughly 25% lower than the other formulations, they displayed substantial *in vivo* gastric residence of greater than 3 days.

**Table 1:** Summary of the three-point bending mechanical testing for solid-arm dosage forms.

	Average Max Flexural Stress (MPa) (N = 3)	Strain Percent @ Max Flexural Stress (mm/mm) (N = 3)
PCL 45k	37.06 ± 3.04	15.77 ± 1.13
MELOX_2	40.12 ± 2.12	17.00 ± 0.67
MELOX_3	30.50 ± 0.59	9.28 ± 0.11
MELOX_5	27.47 ± 2.08	7.17 ± 1.08

### Drug release from v-shaped arms

To uncouple the drug release capacity of the systems from the structural and mechanical aspects we generated arms with a separate compartment for holding the meloxicam formulation. We generated meloxicam-loaded poly(ethylene glycol) and poly(adipic anhydride) reservoirs and placed them into the *v-shaped arms* (formulations described in Fig. 2D). Drug release from poly(ethylene glycol)-based matrices was rapid and nearly 100% of the drug was released within one day of the commencement of the study. Three different molecular weights of the polyether were tested (10, 35 and 100 kDa). Molecular weight did not have any effect on drug release. Drug release from the poly(adipic anhydride)-based arms was significantly slower. About 40% of the drug was released over five days (Fig. 2E).

Our release studies indicated that a range of drug release profiles could be obtained by altering the polymer matrix and by the addition of different excipients. This ability to tune drug release was available with both designs of the dosage form viz. *solid arms* and *v-shaped arms*.

### Extended gastric residence of dosage forms in rabbits and pigs

A central challenge to achieving sustained drug delivery from the gastrointestinal tract is the limited residence of the dosage form in the body. To determine if our systems could be retained in the gastric cavity for extended periods, we administered dosage forms with radiopaque metal beads to animals and at various times monitored their location using radiography. These studies were carried out with two of the three variations of the small and large animal dosage forms in rabbits and pigs, the 21 mm and 46 mm designs.

In pigs treated with the solid arm dosage forms, nearly 90% of the dosage forms (out of n = 15) were retained for 3 days. The *v-shaped arm dosage forms* also showed promising retention, with no failure observed, although only three of these systems were evaluated (Fig. 3A). A representative radiograph of the dosage form residing in the stomach of a pig is shown in Fig. 3C.

Similar results were obtained in rabbits. PCL-based solid arm dosage forms (PCL/PCL) were retained with an efficiency of ~90% over nine experiments. The dosage forms comprised of a polyurethane core and PCL arms (PCL/PU) showed even better retention characteristics. All the dosage forms were retained in the stomach of the rabbits for three days (n = 14). A radiograph of a dosage form resident in a rabbit’s stomach is shown in Fig. 3D.

These results indicated that our systems ranging from 21 mm to 46 mm in diameter and having different designs were capable of residing in the stomach of a representative small and large animal respectively for at least three days. All animals given these dosage forms continued to grow and gain weight as expected and no changes were seen in food or water consumption.

### Plasma pharmacokinetics of meloxicam delivered using the gastric resident dosage form

**Figure 4:**
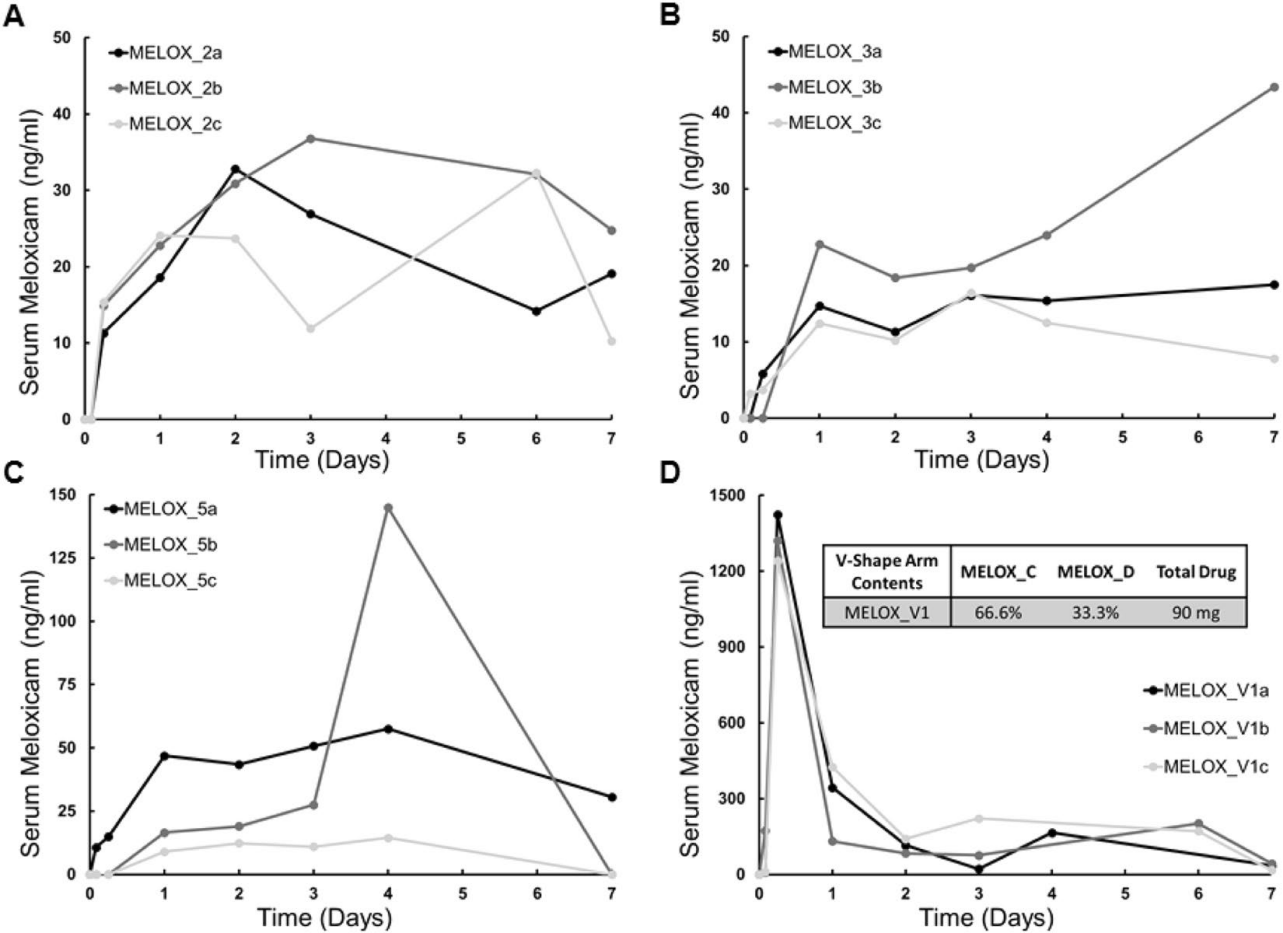
*In vivo* release kinetics of meloxicam in pigs. (**A**) Meloxicam serum concentration over 7 days after single administration of formulation MELOX_2 to pigs. (**B**) Meloxicam serum concentration over 7 days after single administration of formulation MELOX_3 to pigs. (**C**) Meloxicam serum concentration over 7 days after single administration of formulation MELOX_5 to pigs. (**D**) Meloxicam serum concentration over 7 days after single administration of MELOX_V1, a combination of formulations MELOX_C and MELOX_D to pigs; inset shows the composition, see methods section for more detail. See methods section for formulation details and Fig. 2B,D. Each line a, b, and c in each panel represents one animal.

Meloxicam loaded in different formulations either in the solid arm dosage form or the *v-shaped arm dosage form* was administered to pigs. The plasma concentration of meloxicam was monitored over 7 days. When meloxicam was dosed in a solid arm dosage form in a matrix consisting of PCL and poly(ethylene glycol) (MELOX_2), a slow absorption of drug was observed (Fig. 4A). Average maximum concentration of ~30 ng/mL was achieved in 2 days. Interestingly, nearly constant levels of the drug were observed on days 3 and 7 post dose.

In the case where the matrix comprised of PCL, poly(ethylene glycol) and Soluplus (MELOX_3), similar results were obtained (Fig. 4B). The concentrations achieved with this formulation were comparable to those achieved with MELOX_2, and these concentrations were maintained for up to 7 days. These results are in agreement with our i*n vitro* analysis, in that release from MELOX_2 and MELOX_3 were nearly identical.

Plasma concentration of meloxicam when dosed in *solid arm* dosage forms containing PCL, poly(ethylene glycol) and Kollidon CL (MELOX_5) is shown in Fig. 4C. There was high inter-animal variability observed in animals treated with this formulation. Although this formulation led to higher in vitro drug release than MELOX_2 and MELOX_3, plasma levels achieved with this treatment were comparable between these formulations.

Finally, we tested *in vivo* pharmacokinetics of meloxicam dosed using the *v-shaped arms* dosage form (Fig. 4D). A combination of formulations (MELOX_C and MELOX_D, referred to as MELOX_VI) were loaded onto the dosage form. Treatment with this formulation resulted in rapid drug absorption, and a maximum average concentration of 1.3 µg/mL was achieved in 6 hours. On days 1, 2, and 3, average drug concentrations higher than 100 ng/mL were measured. Average serum concentrations at the 6 hr, 1 day, 2 day and 3 day time points for the tabulated drug as well as all formulations tested *in vivo* are shown in Table 2.

**Table 2:** Overview of *in vivo* release in pigs.

Formulation	Delivery Vehicle and Dosage (mg)	6 Hour Serum Concentration (ng/ml)	Day 1 Serum Concentration (ng/ml)	Day 2 Serum Concentration (ng/ml)	Day 3 Serum Concentration (ng/ml)
Meloxicam	Tablet 15	424.4 ± 117.2	58.4 ± 32.9	1.0 ± 1.4	0.0 ± 0
MELOX_2	Solid Arm 90	13.9 ± 1.8	21.8 ± 2.3	29.1 ± 3.9	25.2 ± 10.2
MELOX_3	Solid Arm 180	3.2 ± 2.4	16.6 ± 4.5	13.3 ± 3.6	17.4 ± 1.6
MELOX_5	Solid Arm 180	5.0 ± 6.9	24.1 ± 16.4	24.9 ± 13.4	29.8 ± 16.4
MELOX_V1	V-shape Arm 90	1,328.6 ± 75.4	300.2 ± 123.7	113.6 ± 23.3	106.7 ± 84.5

## Discussion

We have described a scalable dosage form that can be modified to accommodate a range of sizes of species. This is important when considering the variation in size and shape of the gastric cavity across species. In addition, gastric resident systems need to safely transit the gastrointestinal tract without causing trauma or obstruction. We investigated that this prototype dosage form is compatible *in vivo* using two species. In the rabbit, we evaluated the 4 armed, 21 mm diameter dosage form that was placed directly into the gastric cavity using an orogastric tube. Eight out of nine rabbits (PCL/PCL) and fourteen out of fourteen (PCL/PU) retained the dosage form for at least 3 days and all passed without complications. This is the first demonstration of a dosage form of this kind that can reside for greater than 3 days and pass uneventfully in a rabbit model. In pigs, six armed, 46 mm diameter dosage forms were administered in a capsule via an endoscope. Fourteen out of fifteen pigs (solid arm dosage forms) and three out of three (*v-shaped arm dosage form*) retained the dosage form for at least 3 days and no complications were associated with passage of the dosage forms from the gastrointestinal tract. One significant limitation of the current study was that we did not evaluate the stomach post-mortem or histologically to assess signs of toxicity or mechanical damage to the mucosa which will be included for future studies. From our previous work with similar dosage forms in pigs, we did evaluate the gastric mucosa using endoscopy and did not see any visible signs of damage. Regardless, in both rabbits and pigs, they maintained/gained weight, continued to eat well, and had normal fecal output throughout the study period.

The importance of retention for greater than 72 hours in rabbits and pigs was of interest in terms of administering analgesics that would have a duration of action over that time. This would have the potential to negate the need to administer injectable formulations or repeatedly administering the formulation orally. Since meloxicam is available as a sustained release injectable formulation^37^ for rabbits, this could be an alternative oral delivery form. In pigs, these dosage forms could be delivered orally during a procedure or under sedation and provide analgesia for at least 72 hours. It has been shown that oral administration of meloxicam in swine at 0.5 mg/kg, the recommended dose, is well absorbed with a mean peak plasma concentration of 1070 ng/mL with a bioavailability of 87%^25^. In the pigs, we loaded the dosage forms with meloxicam and saw that the amount of drug release can be tuned with varying formulation. The MELOX_V1 formulation showed the most promising release profile of the drug comparable to previous published results^25^. The high inter-animal variability in the pharmacokinetics likely arises from the manufacturing techniques used in this paper. Although some aspects of the dosage form manufacturing are mechanized, significant portion of the manufacturing is still done by hand. Scaling to larger operations will allow us to mechanize all aspects of manufacturing and improve on this variability.

One potential limitation is the dosage form could obstruct the gastrointestinal tract or need to be removed quickly. Our lab has developed methods to add components to dosage forms that allow a triggerable degradation of the dosage form to ensure safe passage and to facilitate removal of the dosage form as this is a potential concern. To ensure safe passage and to facilitate removal of the dosage forms from the animals, we have described two potential solutions before. First, we have described tough polymers that can be used as triggerable linkers in the dosage form. These polymers are tough in the stomach and can withstand the peristaltic forces of the stomach, but upon consumption of liquid “triggering” molecules, the polymer weakens and dissolves. We foresee using polymers like this in the dosage form to connect the central elastomer to the rigid arms. Upon triggering of these dosage forms the various components would fall apart and safely pass from the gastrointestinal tract. We have additionally shown the development of pH sensitive linkers in the dosage forms. These linkers dissolve once the dosage form passes into the intestine. This can serve not only as a safety mechanism but also as a way for safe passage^17,18,38^.

The drug concentrations achieved in the plasma with the MELOX_2, MELOX_3 and MELOX_5 are below serum target levels for meloxicam. Additionally, with the V-shaped arms, we achieved excessive exposures at early time points. Further research is warranted to alter drug exposure with these dosage forms. In theory, increase in drug exposure could be achieved by addition of many hydrophilic polymers in the drug matrices. Moreover, the burst associated with the formulations could be modulated by coating the dosage forms. Any such optimization will require significant *in vivo* validation. *In vitro* experiments provide information that is useful to compare various formulations, but provide minimal guidance on the absolute drug release from each of the formulations *in vivo*. This may be because *in vitro* experiments do not account for the complexity of the gastric fluid, mechanical forces in the stomach and diurnal changes in the gastric environment. Hence, these studies also highlight the need for developing novel methods for developing *in vitro-in vivo* correlation for gastric resident drug delivery systems.

The use of drugs in farm animals should be done with utmost care for ecotoxicity that may arise from excretion of the unabsorbed drug fraction. In our studies, it was difficult to estimate the fraction of the drug unabsorbed from the immediate and sustained release dosage form, especially in pigs due to coprophagia and co-housing with other pigs that had not received the dosage form. A more detailed study analyzing the mechanism of drug absorption may be required in other animal species to determine the fraction of drug remaining unabsorbed from both dosage forms.

The advantage of these scalable systems is that they can be tuned to species-specific characteristics, can be present for a minimum of three days, can pass without complications, and can deliver a variety of drugs that require a prolonged exposure as exemplified in the current study with meloxicam and previously^16^ with ivermectin, a commonly used anti-parasitic drug. Meloxicam is a useful drug in veterinary medicine for analgesia and therefore a useful drug to administer for 3 days, after a painful procedure. At an oral dose of 0.5 mg/kg, it has been shown that mean peak plasma concentration is 1070 ng/ml in mature swine^25^ whereas we were able to show a peak of 1300 ng/ml. Meloxicam at 0.4 mg/kg has been shown to produce some postoperative analgesia after surgical castration of young piglet^39^ and at 1 mg/kg has provided analgesia in sows after lameness induction^40^. The drug release study in pigs shows promise, though future tailoring of the release kinetics will be required for successful translation. Although there are limitations to this system related to the need to formulate the dosage form for each potential drug, the ability to scale a system as well as the potential to combine many different types of therapeutics has tremendous advantages to drug therapy regimens in both humans and animals.

In addition to the applications outlined above, these dosage forms could be applied in other veterinary medical settings for the treatment of chronic conditions in cats and dogs, for example. Chronic medical conditions that require daily administration of multiple drugs could be amenable to these systems providing more consistent treatment and less stressful handling and administration for owners and their pets. In a private practice setting, cost is also a considerable factor to consider when proposing these kinds of systems. The materials and methods used for manufacturing of these systems are readily available, inexpensive and applied for mass production of a broad range of medical and non-medical systems. Although we do not have an exact cost for the dosage form today we anticipate that economies of scale will likely make this a competitive product.

The ability to achieve gastric residence and thereby enable sustained drug levels across different species may have a significant impact in the ability to more effectively treat disorders of animals in research, agricultural, zoological, and clinical practice settings. In addition, these species can be used as models to develop these systems for human applications.

## Electronic supplementary material

Supplementary Information
