# Supplementary material for: Scalable Gastric Resident Systems for Veterinary Application
Source: Sci Rep. 2018 Aug 7;8:11816. doi: 10.1038/s41598-018-30212-3 (PMC6081402; doi:10.1038/s41598-018-30212-3)
Supplement: Supplementary file 1 — Supplementary Information [file 41598_2018_30212_MOESM1_ESM.pdf]

Scalable Gastric Resident Systems for Veterinary Application

Authors: Alison Hayward*[abe], Taylor Bensel*[ab], Hormoz Mazdiyasni[ab], Jaimie Rogner[ab], Ameya Kirtane[ab], Young-Ah Lucy Lee[ab], Tiffany Hua[ab], Ambika Bajpayee[f], Joy Collins[ab], Shane McDonnell[ab], Cody Cleveland[abc], Aaron Lopes[ab], Aniket Wahane[ab], Robert Langer[abd#], Giovanni Traverso[abc#]

[a]The David H. Koch Institute for Integrative Cancer Research, Massachusetts Institute of Technology, Cambridge, Massachusetts 02139

[b]Department of Chemical Engineering, Massachusetts Institute of Technology, Cambridge, Massachusetts 02139

[c]Division of Gastroenterology, Brigham and Women's Hospital, Harvard Medical School, Boston, Massachusetts 02115

[d]Institute for Medical Engineering and Science, Massachusetts Institute of Technology, Cambridge, Massachusetts 02139

[e]Division of Comparative Medicine, Massachusetts Institute of Technology, Cambridge, Massachusetts 02139

[f]Department of Bioengineering, Northeastern University, Boston, Massachusetts 02115

*These authors contributed equally to this work

#Correspondence should be addressed to: Email: ctraverso@partners.org (G. T.); rlanger@mit.edu (R. L.)

Key words: Gastric residence systems, pig, rabbit, meloxicam

Supplemental figure

Table S1. Summary of animal numbers outlining retention and PK groups

**In vivo groups**	**No/group**
**Pigs – retention and PK data**	
Dosage form, no drug	3
MELOX_2	3
MELOX_3 (microcompounded)	3
MELOX_3 (hand mixed)	3
MELOX_5	3
MELOX_V1	3
**Rabbits – retention data**	
PCL/PCL	9
PCL/PU	14
